# Restrictive Versus Massive Fluid Resuscitation Strategy (REFILL study), influence on blood loss and hemostatic parameters in obstetric hemorrhage: study protocol for a randomized controlled trial

**DOI:** 10.1186/s13063-018-2512-z

**Published:** 2018-03-06

**Authors:** Natascha de Lange, Pim Schol, Marcus Lancé, Mallory Woiski, Josje Langenveld, Robbert Rijnders, Luc Smits, Martine Wassen, Yvonne Henskens, Hubertina Scheepers

**Affiliations:** 1 Department of Obstetrics and Gynecology, University Medical Centre Groningen, P.O. 11120, 9700 CC Groningen, the Netherlands; 2 Department of Obstetrics and Gynecology, Maastricht University Medical Centre, P.O. 616, 6200 MD Maastricht, the Netherlands; 3 Department of Anesthesiology and Pain Treatment, Department of Intensive Care, Hamad Medical Corporation, P.O. Box 3050, Doha, Qatar; 4 Department of Obstetrics and Gynecology, Radboud University Medical Centre, P.O. 9101, 6500 HB Nijmegen, the Netherlands; 5 Department of Obstetrics and Gynecology, Zuyderland, P.O. 5500, 6130 MB Sittard-Geleen, the Netherlands; 6 Department of Obstetrics and Gynecology, Jeroen Bosch Hospital, P.O. 90153, 5200 ME ‘s-Hertogenbosch, the Netherlands; 7 Department of Epidemiology, Caphri School for Public Health and Primary Care, P.O. 616, 6200 MD Maastricht, the Netherlands; 8 Central Diagnostics Laboratory, Maastricht University Medical Centre, P.O. 5500, 6202 AZ Maastricht, the Netherlands; 9 GROW: School for Oncology and Developmental Biology and Department of Obstetrics and Gynecology, Maastricht University Medical Centre, P.O. 616, 6200 MD Maastricht, the Netherlands

**Keywords:** Postpartum hemorrhage, Randomized controlled trial, Restrictive fluid resuscitation, Liberal fluid resuscitation, Hemostatic parameters

## Abstract

**Background:**

Postpartum hemorrhage (PPH) is associated with maternal morbidity and mortality and has an increasing incidence in high-resource countries, despite dissemination of guidelines, introduction of skills training, and correction for risk factors. Current guidelines advise the administration, as fluid resuscitation, of almost twice the amount of blood lost. This advice is not evidence-based and could potentially harm patients.

**Methods:**

All women attending the outpatient clinic who are eligible will be informed of the study; oral and written informed consent will be obtained. Where there is more than 500 ml blood loss and ongoing bleeding, patients will be randomized to care as usual, fluid resuscitation with 1.5–2 times the amount of blood loss or fluid resuscitation with 0.75–1.0 times the blood loss. Blood loss will be assessed by weighing all draping. A blood sample, for determining hemoglobin concentration, hematocrit, thrombocyte concentration, and conventional coagulation parameters will be taken at the start of the study, after 60 min, and 12–18 h after delivery. In a subgroup of women, additional thromboelastometric parameters will be obtained.

**Discussion:**

Our hypothesis is that massive fluid administration might lead to a progression of bleeding due to secondary coagulation disorders. In non-pregnant individuals with massive blood loss, restrictive fluid management has been shown to prevent a progression to dilution coagulopathy. These data, however, cannot be extrapolated to women in labor.

Our objective is to compare both resuscitation protocols in women with early, mild PPH (blood loss 500–750 ml) and ongoing bleeding, taking as primary outcome measure the progression to severe PPH (blood loss > 1000 ml).

**Trial registration:**

Netherlands Trial Register, NTR 3789. Registered on 11 January 2013.

**Electronic supplementary material:**

The online version of this article (10.1186/s13063-018-2512-z) contains supplementary material, which is available to authorized users.

## Background

Postpartum hemorrhage (PPH) is the main cause of maternal death worldwide and the main cause of severe maternal morbidity in the Netherlands and other high-resource countries. It is defined by the World Health Organization as blood loss more than 500 ml in the first 24 h after childbirth [1]. Annually in the Netherlands, more than 12,000 cases with more than 1000 ml of blood loss are reported; in about 750 cases, more than four units of packed cells, intensive care admittance or extensive surgical intervention is needed [2]. In total, 33.7% of all women in labor will have more than 500 ml blood loss, 5–13% [3, 4] of whom will experience blood loss of more than 1000 ml and an additional 24.3% of whom will have blood loss of 500 to 1000 ml [3].

Recent publications have shown an increasing trend in PPH in different high-resource countries over the past years [5]. This increase is not directly linked to an increase in women with risk factors for PPH. Two retrospective studies, performed in Australia and Canada, concluded that although the frequency of risk and protective factors for PPH changed during the study period, correction for these factors did not alter the increasing trends in PPH [6, 7].

This increasing trend in PPH is also observed in the Netherlands, despite the introduction of national measures to improve care for this population [4, 8].

Despite the implementation of guidelines, regular training, and obligatory courses, the incidence of PPH is still rising, which is all the more reason to evaluate currently advised protocols for managing PPH. Conclusive evidence for optimal hemostatic resuscitation in PPH is lacking [9]. The Managing Obstetric Emergencies and Trauma course and the Royal College of Obstetricians and Gynaecologists instructions advise generous volume resuscitation to restore blood volume and oxygen carrying capacity: about twice the lost volume and up to 3.5 l of fast fluid infusion in patients with unstable bleeding [10, 11]. The Dutch guidelines advise to start volume resuscitation when there is profuse blood loss; the specific amount is not quantified. This guideline is based on the same (animal) studies mentioned later in this protocol [12]. Volume resuscitation can be done with crystalloids, colloids, or red blood cells in different volume strategies, which all have advantages and disadvantages (see the discussion for a more detailed outline). Our hypothesis is that massive fluid administration might lead to a progression of bleeding due to secondary coagulation disorders in women with PPH.

## Methods and design

### Aims

The aim of the Restrictive Versus Massive Fluid Resuscitation Strategy (REFILL) study is to determine whether in women with early, mild PPH (blood loss 500–750 ml) and ongoing blood loss, restrictive fluid resuscitation strategy reduces progression to severe PPH (defined as blood loss > 1000 ml) compared with care as usual. We hypothesize that restrictive fluid resuscitation will lead to a decrease in progression to severe PPH and therefore a decrease in its adverse outcomes.

### Participant’s criteria and recruitment

In this multicenter study, women with 500–750 ml blood loss postpartum and ongoing bleeding will be eligible for the study. The study will be performed in three Dutch hospitals, two university hospitals (Maastricht University Medical Center, Radboud University Medical Center) and one regional teaching hospital (Zuyderland Medical Center). The Maastricht Medical Center is the coordinating center.

**Fig. 1:**
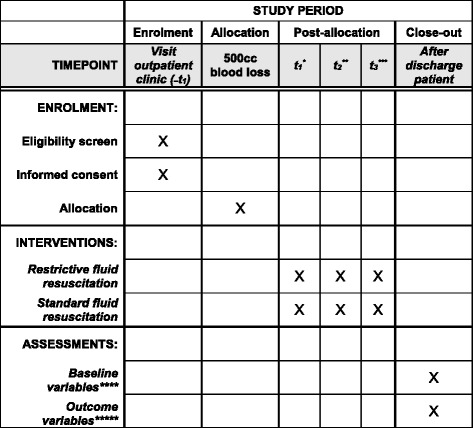
SPIRIT flow diagram. *t_1_ at 500–750 cm^3^, resuscitation within randomized protocol starts, blood withdrawal. **t_2_, 45–60 min after *t*_1_, second blood withdrawal. ***t_3_ 12–18 h after *t*_1_, third blood withdrawal. **** i.e. maternal age, ethnic background, parity (nulliparous or multiparous), gestational age, obstetric history, length, weight, use of oxytocin, mode of delivery (vaginal delivery, instrumental delivery, or Cesarean section), delivery of placenta (spontaneous or manual), life birth, and birth weight. ***** All different treatments to resolve the underlying cause of PPH given to the patient will be registered, intensive care admittance, the need of four or more units of packed cells, embolization, and hysterectomy, laboratory results at *t*_1_, *t*_2_, and *t*_3_

All women attending the outpatient clinic or admitted to the ward and not in active labor who meet the inclusion criteria (see further) will be informed about the study by the treating physician or research nurse. Oral and written informed consent will be obtained. When women present at the labor ward, they will be asked to confirm orally whether they still want to participate in the study (see Fig. 1).

Inclusion criteria are:

- Pregnant and labor starting after 24 + 0 weeks
- Age ≥ 18 years

### Informed consent

- Mentally competent, understanding Dutch language

Exclusion criteria are:

- Prophylactic or therapeutic anticoagulant therapy (carbasalate calcium within the previous 10 days or low molecular weight heparins within previous 48 h)
- Known congenital coagulation disorders
- Pre-eclampsia (higher risk of low plasma volume, higher risk of volume overload)
- Antenatal diagnosis of placenta accreta, increta, or percreta
- Contraindication for massive fluid therapy (e.g. cardiac causes, systemic causes (Marfan), renal causes, pulmonary failure)

### Randomization, procedures, and collection of data

In women with more than 500 ml blood loss and ongoing blood loss, randomization takes place. Treatment allocation is blinded by use of opaque and sealed envelopes. The randomization is stratified per center, in blocks of four and concealed, in an allocation of 1:1. The envelopes will be distributed per center by Maastricht University Medical Center. The required randomization envelopes will be quickly and easily accessible at the labor ward. Participants will be randomized to either the intervention group (receiving fluids at 0.75–1.0 times the blood loss) or the control group, who will receive care as usual (receiving fluids at 1.5–2.0× blood loss).

In women participating in the study, blood loss will be measured by weighing the absorption towels after childbirth, excluding the first one directly after giving birth, as this will include amniotic fluid. In current care, generous volume resuscitation is standard; this consists of about twice the lost volume and 2 l fast infusion in patients with unstable bleeding. Volume resuscitation will primarily be done with a fast infusion of crystalloids or Ringers lactate. In all women, the first 2000 ml will consist of a fast infusion of NaCl (0.9%) or Ringers lactate, or a combination of the two.

At the stage of 500–750 ml blood loss, the study protocol starts (*t*_1_). Intravenous access will be established and a blood sample taken for testing hemoglobin concentration, hematocrit, platelet count, activated partial thromboplastin time, prothrombin time, and fibrinogen concentration. Women delivering in the Maastricht University Medical Centre ROTEM® analysis (FIBTEM, APTEM, INTEM, EXTEM) will be included. Hemodynamic parameters include blood pressure and oxygen saturation, as measured using continuous pulse oximetry.

Additional clinical parameters will guide management and serve as a safety check. We aim to maintain systolic blood pressure > 90 mmHg and diastolic blood pressure > 50 mmHg, or a decrease of less than 20 mmHg. The maternal heart rate should be less than 125 beats per minute [13]. If these cut-off values are crossed, an additional volume of 500 ml will be administered in 15 min, in both groups.

At *t*_2_, 45–60 min after the initial start of infusion, two situations can occur:

1. The patient is stable, defined as normal ongoing blood loss (< 1 full sanitary pad/h) with stable blood pressure and pulse. At this point, we will take an extra blood sample, to test for hemoglobin concentration, hematocrit, platelets, activated partial thromboplastin time, prothrombin time, and fibrinogen concentration.
2. The patient is still bleeding, defined as > 100 ml/h. If the blood loss is greater than one full sanitary pad per hour, the blood loss will be weighed again. Laboratory tests for hemoglobin concentration, hematocrit, and coagulation status will be done, as part of regular care.

At *t*_3_, 12–18 h postpartum, the last blood sample will be taken for hemoglobin concentration and hematocrit analysis. This is part of regular care in women with blood loss > 500 ml.

In the case of > 1500 ml blood loss, the study protocol will be terminated and patients will be treated according to local massive hemorrhage protocol. Blood samples will still be drawn and the patient will be analyzed on an intention-to-treat basis.

Except for the fluid resuscitation, treatment of the underlying cause of the PPH will be according to the local and national protocol (Nederlandse Vereniging voor Obstetrie en Gynaecologie (NVOG) guidelines) in both groups, which will be noted in the clinical chart and registered in the trial data. We expect this to be similar in both groups. The NVOG guidelines advise basal preventive measures to identify women at high risk, consisting of: an active third stage of labor consisting of pre-labor use of an intravenous access and recently known and matched blood type, weighing the amount of blood loss when the blood loss seems profuse and a preventive administration of 5 IU of oxytocin intravenously after childbirth and before placental birth. In women at high risk of PPH, an additional 10 IU oxytocin is administered in a course of 4 h postpartum. The guidelines do yet not recommend tranexamic acid in a preventive setting. If oxytocin is ineffective, or only partially effective, sulprostone (500 μg in 30 min followed by 60–120 μg/h) or methylergometrine (0.2 mg intravenously or intramuscular) is recommended. If blood loss is more than 1000 ml or if more than 2000 ml crystalloids are given, the guidelines advise blood testing (activated partial thromboplastin time, prothrombin time, thrombocyte concentration, fibrinogen concentration, or thromboelastometry, if available) and correct deficiencies accordingly. In expectation of the laboratory results, fibrinogen or tranexamic acid may be administered. This recommendation dates from after the start of the study.

Other study parameters regarding obstetric history and the current pregnancy will be collected from the patient’s chart.

All data are collected and stored anonymously in Maastricht Medical Center in a restricted access file. A trial number assigned to each patient will be used in the dataset to ensure anonymous data collection. These trial numbers are stored securely and locked from the dataset. Data will be imputed as soon as possible after study participation. The dataset will be saved; data will be marked separately by date of saving. Data will be stored for 15 years. PS, NL, HS, and LS will have access to final dataset.

### Outcome measures

#### Primary objective

The primary objective is to establish whether in women with early, mild PPH (blood loss 500–750 ml) a fluid resuscitation strategy with fluids 0.75–1.0 times the blood loss reduces the progression to severe PPH (defined as blood loss > 1000 ml), compared with fluid resuscitation with fluids 1.5–2.0 times the blood loss.

#### Secondary objective

Secondary outcomes are: difference in hemoglobin concentration (mmol/l) 12–18 h postpartum (including differences in hemoglobin < 5.0 mmol/l), differences in transfusion requirements (defined as the number of units of packed red blood cells, fresh frozen plasma, thrombocytes, and fibrinogen needed), differences in the amount of coagulopathies, defined as individually abnormal laboratory results according to current treatment protocols (meaning platelets < 50 × 10,9 fibrinogen concentration < 1 g/L and activated partial thromboplastin time and prothrombin time > 1.5 × mean control).

Severe adverse outcomes will be registered. We define serious adverse outcomes as intensive care admittance, the need of four or more units of packed cells, embolization, and hysterectomy [2].

### Statistical analysis

The between-group difference in the proportion of women progressing from early mild PPH to severe PPH and its confidence interval will be calculated. Descriptive analysis will be carried out for baseline characteristics, i.e. maternal age, ethnic background, parity (nulliparous or multiparous), gestational age, obstetric history, length, weight, use of oxytocin, mode of delivery (vaginal delivery, instrumental delivery, or Cesarean section), delivery of placenta (spontaneously or manual), live birth, and birth weight. All different treatments to resolve the underlying cause of PPH given to the patient will be registered. Severity parameters will be described, i.e. intensive care admittance, the need of four or more units of packed cells, embolization, or hysterectomy.

Total blood loss, transfusion need, and laboratory results will be compared by use of either Student’s *t* test for continuous outcomes or the chi-square test for dichotomous outcomes. In the case of non-normality, mathematical transformation will be carried out of continuous outcomes. In the case of large differences in important prognostic variables at baseline (which are unanticipated in view of the randomization), multivariable logistic or linear regression analysis will employed, to control for these variables. Analysis will be by intention to treat. Missing data will not be imputed by use of multiple imputation. All data will be analyzed using IBM SPSS 24.0 software.

### Sample size calculation

With the current standard of care, about 30% of all women will proceed from 500 ml to 1000 ml of blood loss. With a reduction from 30% to 15% (beta 0.80, alpha 0.05), 2 × 118 (236) women will have to be included. We aim to include 250 women, to compensate for loss to follow up or incomplete data.

### Safety concerns

A data safety monitoring board has been established to perform ongoing safety surveillance and interim analyses of the safety data. The board will be informed in any case of a severe adverse event. The board is composed of three independent physicians: Dr. NMAA Engels (anesthetist), Dr. JM Middeldorp (gynecologist from a hospital not involved in the trial), and Dr. A Kessels (epidemiologist). Dr. NMAA Engels is chairman of the board. Further details about the board are listed in a separate charter, which is available on request.

The data safety monitoring board will meet by teleconference after the first 2 × 25 patients have been treated and after every 50 patients thereafter per group, and will conduct an interim analysis on the primary objective measure and the composite measure of severe outcome events (maternal death, use of more than four units of packed cells, intensive care admittance, embolization, or operative intervention.) The formulas proposed by Prochan, Lan and Wittes [14] will be used for the interim analysis.

In the analysis conducted by the data safety monitoring board, a correction will be made for possible confounders for the primary outcome, such as risk factors for PPH and difference in combined severe outcome. Should there be a statistical significant difference in severe adverse events between the intervention and the control group that cannot be accounted to other factors, such as selection bias in small groups, the data safety monitoring board shall decide whether the study should be continued. The study could be terminated prematurely on the advice of the data safety monitoring board if one of the treatment protocols shows less progression to severe blood loss and less maternal morbidity; in this case, it is not ethically justified to continue the study. All communications by the data safety monitoring board are reported via email to HS and PS. The final decision to terminate the trial on the advice of the data safety monitoring board lies with HS.

The trial is overseen by PS and HS. They are responsible for all communications with participating hospitals and local research nurses, and are also responsible for communication with the data safety monitoring board, the ethics committee, and the trial registration bureau.

Insurance policies are available to patients in case of adverse outcomes with lasting effects due to the study intervention.

## Discussion

Postpartum hemorrhage is increasing in incidence in industrialized countries; there is no evidence-based managing protocol regarding fluid resuscitation. Volume resuscitation can be done with crystalloids, colloids, or red blood cells, which all have advantages and disadvantages. Resuscitation with crystalloid fluids means that large amounts are needed, which may induce acidosis and coagulopathy, the formation of interstitial edema and impairment of the microcirculation [15]. Colloid fluids, in particular synthetic colloids, such as hydroxyethyl starch solutions, may impair clot formation and therefore increase blood loss [16, 17]. Furthermore, even new-generation medium molecular weight hydroxyethyl starch solutions disturb fibrin polymerization in patients undergoing spine surgery [18]; the presence of hydroxyethyl starch solutions or gelatin solutions in patients with fibrinolysis leads to faster clot disintegration [19]. Once 30–40% of the circulating blood volume is lost, red blood cell replacement will be required. Red blood cells cannot be used for massive fluid therapy and one must be careful not to use uncrossmatched blood, especially in young fertile women, because of the possibility of irregular antibody formation and its effect on future pregnancies.

Restrictive or permissive resuscitation has recently been advocated as an alternative to the current standard care. In animal studies, military settings, and studies of non-pregnant trauma patients, controlled hypotensive resuscitation has been investigated. These studies have shown that there might be an advantage for a restrictive fluid resuscitation strategy. However, there are few well-performed randomized controlled trials. This might be due to ethical concerns in life-threatening conditions, but is nonetheless important to improve survival and morbidity, relying on evidence-based medicine. Until recently, high-volume fluid resuscitation strategies have been used to reverse hemorrhagic shock by replacing blood loss with intravenous fluid or transfusions. This strategy has been the gold standard, even though it has not been tested in prospective randomized clinical trials and has considerable limitations and risks. Increasing evidence has demonstrated that aggressive crystalloid-based resuscitation strategies are associated with cardiac and pulmonary complications, gastro-intestinal dysmotility, coagulation disorders, and immunological and inflammatory mediator dysfunction. Aggressive fluid administration increases arterial and venous pressures, but aggravates the dilution of clotting factors and blood viscosity, which results in increased hemorrhage volume, decreased oxygen delivery, and decreased survival rates [20]. Continued fluid administration and positive fluid balances have not been shown to improve renal outcomes and may worsen overall prognosis in acute kidney failure [21]. Also, maintaining a high or “normal” blood pressure in patients with uncontrolled hemorrhagic shock can result in the “lethal triad” of hypothermia, acidemia, and coagulopathy [22].

Theoretical concerns regarding the safety of restrictive resuscitation are based on the possible harmful effects of decreased oxygen delivery to the various tissues of the body due to shock. Maintaining a blood pressure that is too low could potentially result in inadequate perfusion and subsequent organ failure. Intraoperative restrictive resuscitation has been successfully used in several animal models [23]. The results of Lu *et al.* [24] showed that aggressive fluid resuscitation to restore near-normal mean arterial pressure of 80 mmHg during uncontrolled hemorrhage induced massive blood loss and excessive hemodilution. Controlled fluid resuscitation to maintain mean arterial pressure of 40 mmHg in the presurgical treatment of severe and uncontrolled hemorrhagic shock decreased further blood loss, avoided excessive hemodilution and coagulopathy, improved the early survival rate, and reduced apoptosis of the visceral organs [24].

Two randomized controlled trials investigated restrictive resuscitation protocols in trauma patients. The preliminary results on 90 patients in a randomized controlled trial conducted by Morrison *et al.* [25] showed fewer early postoperative deaths and significantly fewer blood product transfusions in the study group, without differences in the incidence or severity of coagulopathy, thrombocytopenia, or anemia. In this study, the difference in mean arterial pressure between both study groups was not statistically significant and the actual mean arterial pressures for the two groups were much more similar than might be expected based on the target goals for resuscitation [25]. The final results of this trial, including results obtained with the targeted 271 patients, have not been published yet. Dutton *et al.* [26] found no significant difference in mortality between the study groups in a trial in which maintaining a systolic blood pressure > 100 mmHg was compared with targeting a systolic blood pressure of 70 mmHg [26]. One of the limitations of this study is that no power analysis was performed before initiating the study and determining the sample size (*n* = 110) Also, as in the previously mentioned study, the proposed target blood pressure in the study group was not achieved.

A retrospective analysis by Duke *et al.* [27] of trauma patients showed an overall lower mortality rate, lower intraoperative mortality, and a shorter hospital stay in the restrictive resuscitation group. Despite the fact that the groups were well-matched, the retrospective character of the study is a limitation and bias cannot be ruled out [27].

A prospective randomized pilot trial comparing controlled resuscitation versus standard resuscitation in hypotensive trauma patients demonstrated that controlled resuscitation strategy can be successfully and safely implemented in a civilian environment. The results showed a reduction of early crystalloid resuscitation volume, but also an increase in early blood product transfusion [28].

In all, these studies, there were difficulties in following the study protocol strictly. This may be explained by the fact that these studies were performed in an acute setting and by the impossibility of blinding the treating clinicians, who might be less familiar with restrictive resuscitation.

In conclusion, there is little and contradictive evidence for either aggressive or restrictive fluid resuscitation, and it is very difficult to perform good clinical trials. This is reflected in the latest guidelines. The European Society of Anaesthesiology guidelines on management of severe perioperative bleeding recommend avoidance of hypervolemia. Permissive hypotension is not mentioned, but implementation of delayed or low-volume resuscitation protocols is not yet recommended [29]. Conversely, the updated European guidelines on management of bleeding and coagulopathy following major trauma recommend a target systolic blood pressure of 80 to 90 mmHg until major bleeding has been stopped in the initial phase following trauma without brain injury [30].

All these results, mainly obtained for trauma patients, cannot be extrapolated to pregnant women during labor, in view of the physiological hemodynamic and hemostatic changes that occur in pregnancy [31, 32]. Plasma volume increases up to 40% during pregnancy, whereas red blood cell count only increases by 30%, cardiac output is increased, and systolic and diastolic blood pressure decrease slightly in the second trimester and elevate toward term pregnancy. At the end of normal pregnancy, changes in the coagulation and fibrinolytic system result in an apparent hypercoagulable state [33–35] to minimize blood loss at delivery. In patients with PPH, this equilibrium will be altered and may lead to profound and rapid changes in hemostasis. In some cases, the disruption of the coagulation precedes delivery and may contribute significantly to development of PPH [36]. Timely recognition and prompt intervention are crucial for the successful management of PPH [37]. In non-pregnant individuals with massive blood loss, restrictive fluid management has been shown to prevent a progression to dilution coagulopathy [16–19].

In conducting this study, we hope to find the best managing option for treating PPH and to achieve a decrease in adverse outcomes by reducing the severity of PPH.

## Trial status

Protocol version 8, February 2015. Recruitment started August 2014. The trial is ongoing; 179 out of the 250 patients have been enrolled in the study. A planned interim analysis in October 2017 by the data safety monitoring board showed no safety issues needing discontinuation and a high likelihood that the hypothesis will be proven. The trial will end if the 250 patients are reached or when the data safety monitoring board intervenes after planned interim analysis.

## Additional files

Additional file 1: SPIRIT fillable checklist. (DOC 122 kb)

Additional file 2: TIDieR checklist. (PDF 410 kb)

## Abbreviations

NVOG: Nederlandse Vereniging voor Obstetrie en Gynaecologie
PPH: postpartum hemorrhage
REFILL: Restrictive Versus Massive Fluid Resuscitation Strategy
TIDieR: template for intervention description and replication
